# Between the center and periphery: Commodification, formalisation, and infrastructuralisation of grassroots innovation

**DOI:** 10.1371/journal.pone.0290682

**Published:** 2024-01-17

**Authors:** Pengfei Fu, Zhipeng Zang

**Affiliations:** 1 School of Media & Communication, Shanghai Jiao Tong University, Shanghai, China; 2 School of Humanities, Tongji University, Shanghai, China; Universiti Sains Malaysia, MALAYSIA

## Abstract

The question of whether China can become a creative nation has been a topic of much debate in academic circles. The Chinese government has expressed its belief that China can develop a unique form of creativity to move the country from the periphery to the center of the global creative ecosystem. This perspective has led to a series of state-led trials and experiments, including the adoption of cultural and creative industries, creative clusters and cities, and the recent maker movement. This paper utilizes the center-periphery theory to analyze the emergence, development, and evolution of China’s maker movement, aiming to revisit the creativity issues in contemporary China. Based on three years of ethnographic research, the paper unpacks the maker movement at three interrelated levels: individual, organizational, and urban. Empirical data indicates that the transformation of China’s maker movement is characterized by commodification, formalization, and infrastructuralization processes. The tension between growth and development, and stability and control has turned the once grassroots maker movement into a contested creative hybrid. This paper challenges the conventional view that China is resistant to change and incapable of creativity due to institutional and ideological influences. It demonstrates how an alternative mode of creativity can emerge outside global creative centers and proposes a new perspective on China’s potential to become a creative nation.

## Introduction: From CCIs to the maker movement

### From CCIs to digital CCIs: A Chinese tale

It’s been over a decade since the concept of Cultural and Creative Industries (CCIs) arrived in China. In 2005, scholars and policy-makers introduced the concept of CCIs with the aim of transitioning China’s economy from low-value manufacturing industries to high-value creative and innovative economies [1, 2]. The concept of cultural and creative industries (CCIs) has gathered momentum with state backing, coinciding with the rhetorical prioritization of "soft power" by the Chinese government, aimed at enhancing China’s innovative national image and transitioning it from the global creative periphery to the center [3, 4]. The development of CCIs also harbors the potential to reconstruct creative communities by fostering local human resources and drawing in global talents. The construction of creative communities entails Xiaoping’s 1978 dictum ‘the liberation of thought’ [5] and the central governments’ social-economic imaginaries of the construction of ‘modern China’ [6]. However, scholars, such as Keane [7] and Zhang [8], have raised concerns regarding the feasibility of the adoption of CCIs in China’s unique social and cultural contexts. These scholars contend that the purported success of CCIs, as evidenced by impressive statistical data, conceals the potential negative impacts of CCIs, such as gentrification and polarization, particularly in sectors and communities that are deemed "uncreative" [9].

In recent years, the Chinese central government has shifted its focus from the CCIs to the digital cultural and creative industries, highlighting the need to integrate cultural creativity with digital technologies. The digital CCIs were first formally introduced in China in 2008 when the State Council issued the "Guidance on Further Promoting the Reform, Opening-up and Economic and Social Development of the Yangtze River Delta Region". According to this policy document, digital CCIs are understood as an emerging economic model ‘integrating modern information technology and cultural and creative industries’ [10]. The "digital turn" illustrates governments’ aspirations to bolster CCIs through the utilization of digital technologies, including the Internet, 5G, and artificial intelligence. This reflects a search for novel means of fostering growth and development within the sector [11]. Policy roadmaps such as "Internet+," "Made in China 2025," and "Mass Innovation, Mass Entrepreneurship" serve as cornerstones for China’s aspirations of becoming a technologically advanced, innovative, and futuristic nation, ultimately culminating in the achievement of the "Chinese Dream" of national rejuvenation [12, 13].

**Fig 1:**
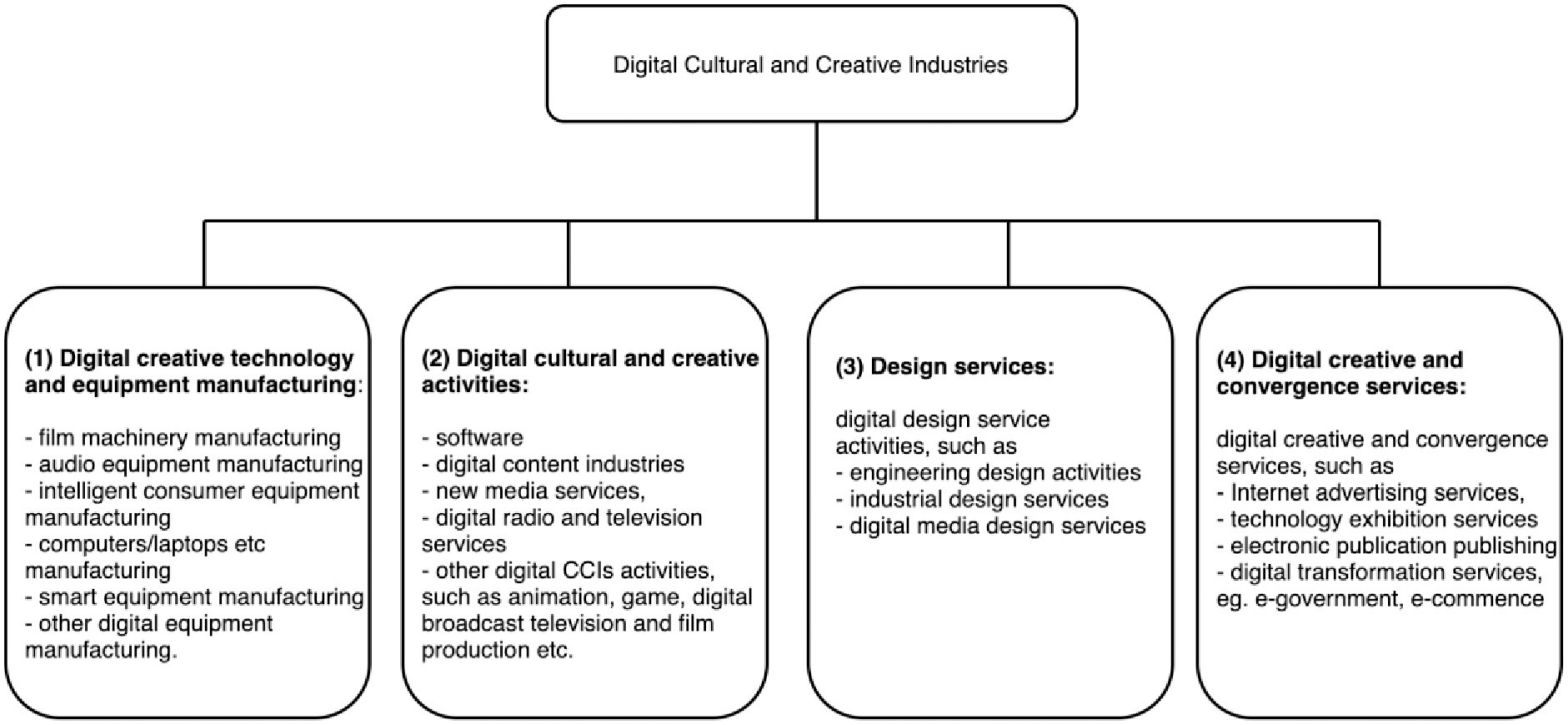
The digital CCIs categories by China’s National Bureau of Statistics.

In 2021, "implementation of cultural industries digitalization strategy" was written into the 14th Five-Year Plan. In May this year, the General Office of the Chinese Communist Party (CPC) and the State Council issued the "Opinions on Promoting the Implementation of the National Cultural Digitalization Strategy", which further emphasized the importance of digitalisation for CCIs [10]. In contrast to previous endeavors in digitalization, China has made significant strides in the development of digital CCIs. Notably, the successful emergence of Internet giants such as Baidu, Alibaba, Tencent, and more recently, the burgeoning platform TikTok, has instilled a sense of confidence in the state to provide further support for these nascent enterprises. According to the National Bureau of Statistics data, in 2021, the national digital CCIs achieved a business income of 396.23 billion RMB (57 billion USD), an increase of 18.9% over the previous year; with a two-year average growth of 20.5% [14]. In contrast, notwithstanding the promising official statistics, there is ongoing debate and contestation surrounding the definition of digital CCIs. Fig 1 below depicts the digital CCIs classification as issued by the National Bureau of Statistics. Despite the variability in the statistical coding for digital CCIs over time, the incorporation of these industries in official categorization reflects the government’s steadfast commitment to advancing its digitalization strategy.

### From the periphery to the center: The rise of the maker movement

The center-periphery model is a spatial metaphor that describes and attempts to explain an unbalanced structural relationship or distribution of power between the advanced "center" and less-advanced "periphery" within a particular country or political, social, and cultural systems [15, 16]. The application of the center-periphery model allows for a comprehensive investigation of the interdependent central and peripheral components of CCIs and their interactive dynamics. It helps us examine the power dynamics between the economically and politically dominant parts of CCIs (the center) and the marginalized or underdeveloped parts of CCIs (the periphery), and explore the underlying mechanisms that have facilitated their persistence or transformation over time.

Policies regarding CCIs have faced criticism for excessively prioritizing emerging high-end cultural and creative businesses and communities while marginalizing activities and workers deemed "uncreative" and "outdated" [17, 18]. The transition from traditional manufacturing industries to CCIs, including digital CCIs, has sparked debates on China’s capacity for creativity within its existing social, cultural, and political framework, as well as what should be the focal point of CCIs [19]. Some scholars suggest reevaluating the discourse on creativity in China to explore alternative forms of creativity in non-Western contexts, while others propose redirecting CCIs research and policy-making to emphasize the material aspects of cultural production, such as small-scale manufacturing [18]. The latter perspective revolves around transforming and relocating these traditional sectors to the center or at least incorporating them into Chinese CCIs. As a global manufacturing powerhouse, China boasts a well-established manufacturing industry. However, repackaging the domestic manufacturing sector and enhancing China’s soft power and international reputation pose significant challenges for the government due to mounting international competition [20].

The global maker movement [21] provides a potential way for China to bridge traditional manufacturing industries and CCIs by using cultural creativity to redesign the relationship between traditional industries and CCIs. In 2015, Premier Li Keqiang launched the "mass innovation, mass entrepreneurship" (MIME) initiative in the government work report, which aims to address employment issues, increase people’s incomes, promote social mobility, fairness, and social justice. On September 18, 2018, the State Council issued the "Opinions on Promoting High-Quality Development of Innovation and Entrepreneurship" to create an upgraded version of "Double Innovation". The updated MIME initiative recognizes innovation as a vital engine for economic and social development in three ways: promoting the integration of new technologies and traditional industries to transform and upgrade traditional industries, accelerating the cultivation of new industries by linking traditional industries with new technologies, and creating an open and innovative environment that enables everyone to participate in grassroots innovation and entrepreneurship, contributing to building an "innovative nation" [22].

However, the question remains: can the top-down MIME initiative truly transform the landscape of CCIs in China? If so, to what extent and in what ways? Previous research has approached this question from different perspectives. Wen [23] investigated the intrinsic link between the maker culture and China’s CCIs, focusing on communities, spaces, activities, policies, and innovation, using a macro lens. Wen argued that the convergence of cultural creativity and technology indicates a shift toward more technology-driven CCIs in China. Bolli [24] examined how individual makers, hackers, and innovators are influenced by China’s cultural contexts and government initiatives from the perspective of individual makers. Fu [25] scrutinized the governance model of three maker communities in China and argued that Chinese makerspaces follow a "subtle top-down" model, which is a state-led, multi-actor governance model.

While previous studies have provided valuable insights on the maker movement in China, there is a need for a holistic view to critically examine its impact on China’s CCIs. This paper aims to address this question by examining the emerging maker movement in China at three levels: individual makers (as innovators), makerspaces (as a new organizational form for innovation and creativity), and maker cities (as a new form of urban CCIs). Our approach is informed by the understanding that innovation is a hybrid process that involves multiple stakeholders across different levels, and we chose these three levels to build our case study with three research questions:

- RQ1: What defines the typical Chinese maker and how do they compare to the creative class?
- RQ2: How does the governance of Chinese makerspaces impact innovation within maker communities?
- RQ3: What is the connection between maker culture and local urban creative and cultural industries, and how can the maker movement contribute to their growth?

## Methodology and data

This study aims to investigate the three layers of the maker movement [26] and their relationship with CCIs in China. To achieve this, a mixed-methods approach was adopted, with ethnography serving as the principal research method and semantic network analysis employed as a complementary quantitative tool. This study was approved by the Science and Technology Ethics Committee of Tongji University, Shanghai, China. The ethnographic methodology [27], which involves a comprehensive and prolonged on-site investigation to comprehend the intricacies of China’s maker movement as a social and cultural entity, was implemented to address RQ1 and RQ3. On the other hand, semantic network analysis was used to respond to RQ2. Informed consent was obtained from all participants for all the studies.

The ethnographic fieldwork was conducted in three major cities in China–Beijing, Shanghai, and Shenzhen–which were chosen for their prominence in CCIs development and maker activities. The fieldwork was conducted from 2017 to 2020, resulting in over 80 hours of interviews and observations, and produced 842 pages (19,674 lines) of transcripts. To analyze this rich data, an ethnographic coding method was applied, following the procedures outlined by Huberman and Miles [28] and LeCompte and Schensul [29, 30]. The coding process involved open coding, axial coding, and selective coding, resulting in the identification of 132 concepts grouped into 30 categories. The categories were integrated into a central paradigm using selective coding, which enabled the identification of relationships between categories and the construction of a theoretical framework based on the findings.

To address RQ2, semantic network analysis was employed to examine the governance of makerspaces and its impact on innovation within maker communities. A self-definitional approach was used to analyze 305 makerspaces, hackerspaces, Fablabs, and mass innovation spaces in China. The analysis focused on five key dimensions: space, members and community, resources, activities, and values/philosophy. Software tools such as Gephi and R were used for data cleaning and network analysis, resulting in a network of 3,527 nodes and 36,892 edges. A frequency analysis was conducted to identify the prominent nodes, representing the core concepts and themes that underpin the self-understanding of makerspaces in China. The resulting top 30 nodes are presented in Table 2, which offers valuable insight into the prevalent themes and characteristics associated with Chinese makerspaces.

Overall, this mixed-methods approach enabled a comprehensive exploration of the maker movement and its relationship with CCIs in China. The rich ethnographic data provided insights into the characteristics of Chinese makers and their impact on local CCIs, while the semantic network analysis shed light on the governance of makerspaces and its implications for innovation. The theoretical framework developed from the findings contributes to theory building in the field of maker culture and CCIs.

## Results

The present study aims to provide a comprehensive understanding of the Chinese maker movement and its relationship with the CCIs. To this end, the analysis is conducted through three distinct sections, each exploring the phenomenon from a unique perspective. The first section examines the Chinese maker movement from the perspective of individuality and collectiveness, critical making, and commercial entrepreneurship. This section seeks to comprehend how the imported ideals of the maker movement have been transformed and adapted by Chinese creators with Chinese characteristics.

The second section focuses on the organizational structure and governance of makerspaces to explore how this newly emerged organization is appropriated by external powers. This section provides insights into the power dynamics at play in the maker movement and the implications of these dynamics for the development of alternative paradigms for CCIs.

The third section investigates the maker movement at the urban level, with the aim of understanding the mechanisms of innovation, culture co-creation, and production between urban CCIs clusters and maker industries. By exploring the interplay between the maker movement and the broader urban context, this section highlights the potential for the maker movement to contribute to the development of more sustainable and inclusive CCIs.

### Commodification: Makers as entrepreneurial creators

We first situate Chinese makers as the central focal point within the growing tensions between critical making and commercial entrepreneurship, as well as between bottom-up grassroots innovation and top-down ‘mass innovation.’ Through a lens of commodification, we aim to examine the intricate struggles and transitions that Chinese makers undergo as they navigate the often-conflicting realms of commercialism and criticality, grassroots autonomous innovation, and state-led collective innovation. In the context of maker culture, commodification can manifest in various forms, such as the commercialization of DIY products or the monetization of makers’ creative output. This process has the potential to conflict with the values of critical making and grassroots innovation, as the pursuit of profit may take precedence over the pursuit of social or environmental good.

Our inquiry into the experiences of Chinese makers specifically delves into the ways in which this imported culture, with its strong emphasis on bottom-up and autonomous innovation, intersects with and evolves within the unique context of China. Through a series of in-depth, semi-structured interviews with 26 makers from diverse demographic backgrounds, we seek to shed light on the challenges and opportunities that arise from the commodification of maker culture in China.

Firstly, our research reveals that a significant proportion of contemporary makers are individuals who were previously marginalized and associated with the "shanzhai" movement in China. The shanzhai culture and movement of the past not only yielded a more so phisticated manufacturing and supply chain, but also fostered a vast number of grassroots practioners with expertise in open-source hardware and software prototyping [31]. Enabled by the shanzhai social fabric and innovation ecosystem [32], shanzhai creators are capable of developing novel products and alternative models of innovation participation and production, while challenging the imbalanced global creative hierarchy [33].

We found that the early Chinese maker culture was profoundly influenced by the shanzhai movement, prioritizing openness, collaboration, peer learning, and the so-called ’grabism’ [14, 34, 35]. Chinese makers of the early era endeavored to subvert the innovation monopoly of international industry giants by spearheading open-source initiatives. Through the critical making lens [36, 37], their practices can be regarded as diverse forms of critical making that underscore the social significance of production.

The state-led mass innovation and mass entrepreneurship initiatives, with a top-down approach, have transformed the role of marginalized Shanzhaiers, positioning them at the forefront of the Creative and Cultural Industries (CCIs) as "makers." Our research reveals that a vast majority of Chinese makers are presently more inclined towards entrepreneurship, emphasizing utility-oriented pursuits. Specifically, they have moved beyond the "gift economy" paradigm, towards a more commodified "entrepreneurial solutionism" paradigm, as elucidated by Keane and Chen [13]. The term "entrepreneurial solutionism" characterizes a tendency to perceive entrepreneurship as a universal remedy for diverse problems. Based on our interview and observation data, we conclude that Chinese makers embrace an "entrepreneurial solutionism" mindset, perceiving entrepreneurship as an effective solution for resolving career development, personal growth, family happiness, and other issues.

Hao exemplifies the transformative shift among Chinese makers. Initially, like many others, he joined a tech company upon returning from studying in the US. However, upon discovering promising opportunities in China, he began developing projects with friends during his spare time. As the projects progressed, Hao made the decision to leave his tech job and become a full-time maker-entrepreneur. In his own words, here is his journey from amateur maker to professional maker-entrepreneur:

*"[*…*] I started out just spending time with my friends*. *But when I realized that there were business opportunities everywhere around me and my friends started becoming entrepreneurs*, *I wondered if I could also turn my hobby into a business*. *Moreover*, *the policies were supportive at the time*, *with innovation and entrepreneurship being widely promoted*, *and a positive societal atmosphere all around [*…*]*.*"*

*(Interviewee Hao, 2019)*

Hao’s testimony highlights the critical role of state policies in enabling the commodification-induced transformation of Chinese makers, facilitating their transition from a subculture of "underground" makers to an integral part of the creative class. This transformation is driven by the impact of the commodified socio-economic environment, as demonstrated by the proliferation of opportunities, supportive policies, and favorable conditions. This environment is shaped by the government’s MIME policy initiatives, reflecting an "entrepreneurial solutionism" perspective that views makers as a key solution to societal and economic challenges and a means to advance China’s aspirations as an innovative nation. The government’s emphasis on promoting the growth and success of makers underscores their importance as a driver of economic and social progress.

Our study reveals that Chinese makers conceive of "making for the state" as a collective endeavor aimed at building an innovative nation, fostering greater creativity, innovation, and advancement. For instance, Wen Li, a maker at Shanghai’s Mushroom Cloud makerspace, has developed a social innovation-oriented project focused on designing and developing maker/STEM education for rural children. However, due to the project’s difficulty in securing market support, he turned to collaborate with the government, leveraging policy and financial assistance to enhance the content and delivery of maker education. Through participation in various government-led outreach initiatives, he successfully expanded the program to schools in the mountainous regions of southwestern China. As he stated:

*[*…*] Initially*, *we intended to produce commercial maker education products*, *but we discovered that the market was oversaturated*, *and the competition was intense*. *However*, *due to the publicity of MIME*, *we decided to contribute by redirecting our project’s focus towards serving underprivileged groups*. *Our goal is to create maker education packages for students in remote areas and make a positive impact on innovative China*. *Ultimately*, *a country’s strength relies on its bottom line rather than its top line*.*"*

*(Interviewee Wen Li, 2020)*

The interviewee’s statements reflect a maker’s mindset centered on nationalistic creation. Further investigation reveals that this collective imaginary is embedded within daily making practices, responding to governmental calls for action. This bears resemblance to previous discourses, such as creative state-led initiatives, the emergence of creative industries as a new modernity [19], and the proliferation of entrepreneurial solutionism and characteristic cultural industries [13, 38, 39]. Individual makers are inextricably linked with broader political discourses and undergo negotiation and transformation, resulting in a new form of maker culture and ideology that differs fundamentally from its western counterparts.

The findings of this study reveal that Chinese makers have incorporated certain Western maker ideologies, while also infusing them with unique Chinese characteristics. The state-led national maker movement in China has established a robust manufacturing infrastructure and governmental backing for product commodification, thereby providing Chinese makers with an advantage over their Western counterparts. The Chinese makers’ discourse on "making for the state" has fostered a distinctive maker ideology and mindset, marked by utilitarianism, entrepreneurialism, technological determinism, and techno-utopianism. They consider making as a means to enhance their personal lives and social status, while also promoting collectivism and institutionalism for the betterment of society and the revitalization of the nation. In essence, Chinese makers have developed a distinctive approach to making that amalgamates Western and Chinese components, leveraging the opportunities presented by the national maker movement to achieve their objectives. The empirical data demonstrates the intricate challenges and transformations that Chinese makers undergo as they navigate the complex domains of commercialism and criticality, grassroots autonomous innovation, and state-led collective innovation.

### Formalisation: From makerspace to mass innovation space

The concept of makerspaces has garnered increasing recognition as a site for grassroots innovation, with scholars heralding them as a promising alternative economic model and educational paradigm [40]. Research has shown that makerspaces in the global south exhibit a range of diverse trajectories, which are shaped by the underlying political, cultural, and social contexts. In the case of China, the national MIME policy blueprint is geared towards the formalization of previously underground "informal communities," with the goal of expanding makerspaces throughout the country.

This section examines the institutionalisation and formalisation of Chinese makerspaces using a semantic network analysis method. We analyze various types of makerspaces to investigate their governance structure, institutionalization and formalisation processes, and the challenges of balancing individual autonomy with collective participation. Empirical data (Table 1) sheds light on the governance and transformation of makerspaces in China and offers insights into the complex interplay between institutionalization, innovation, and culture production. The analysis of our data reveals the presence of three primary modes of maker communities in China: corporate-led incubators, state-led mass innovation spaces, and enthusiast clubs. Table 2 provides a concise summary of these three models of makerspaces.

**Table 1:** Descending list of 30 most frequently appeared nodes and values.

No.	Category	Word/Node	Frequence	Value
1	activities	entrepreneurship	923	3.73%
2	activities	innovation	514	2.08%
3	activities	incubation	352	1.42%
4	resources	provide	331	1.34%
5	resources	platform	264	1.07%
6	space	space	259	1.05%
7	space	mass innovation space	248	1.00%
8	members and community	entrepreneur	213	0.86%
9	members and community	maker	211	0.85%
10	resources	resource	205	0.83%
11	resources	technology	201	0.81%
12	resources	industries	191	0.77%
13	activities	invest	178	0.72%
14	space	incubator	175	0.71%
15	members and community	team	167	0.68%
16	activities	build	146	0.59%
17	activities	development	144	0.58%
18	space	office	141	0.57%
19	resources	operation	124	0.50%
20	space	base	110	0.44%
21	activities	connect	106	0.43%
22	resources	technique	99	0.40%
23	values/philosophy	culture	94	0.38%
24	values/philosophy	creativity	92	0.37%
25	members and community	institution	91	0.37%
26	activities	management	91	0.37%
27	values/philosophy	China	90	0.36%
28	values/philosophy	model	90	0.36%
29	resources	expertise	86	0.35%
30	values/philosophy	smart	85	0.34%

**Table 2:** Three dominant modes of Chinese makerspaces.

	Corporate-led incubator	State-led mass innovation space	Enthusiast clubs
Cases	Mushroom Cloud makerspace, TCL makerspace, Tencent Makerspace	SZU Makerspace, Beidou makerspace, CASS maker institute	SZDIY, Xinchejian
Space	Co-working space, intermediary innovation incubator.	Co-working space	Prototyping workshop
Members and community	Mixed between open bottom-up and closed hierarchical	Traditional and hierarchical	Bottom-up, community self-organization
Resources	Focus on sharing working space	Government funding and support.	Prototyping resources
Activities	Platform-based innovation and production	Circumscribed innovation and production	Commons-based peer production, co-production
Values/philosophy	Innovation co-production, entrepreneurship focused	Collectiveness; making for the state; build an innovative nation	Openness, sharing, learning, grassroots innovation

The corporate-led incubator model of makerspaces, which seeks to augment innovation capabilities and sustainability through leveraging external innovation resources. This approach involves the provision of commercial opportunities and resources, thereby institutionalizing and formalizing makers and their innovations. An example of this model is the makerspace associated with TCL Technology Group, a prominent Chinese electronics company renowned for designing and marketing electrical products such as mobile phones, air conditioning, and televisions. TCL, like other electronics manufacturers, is faced with the challenge of digitalization and the ever-changing dynamics of markets and consumers. The conventional research and development (R&D) approach may be insufficient in maintaining a competitive advantage in the highly contested market environment. As a result, TCL has adopted a strategy of breaking down innovation barriers and tapping into external innovation resources.

To achieve a balance between incorporating external innovation resources and retaining control over innovation production, the establishment of a makerspace presents a pragmatic solution. The makerspace serves as an intermediary platform that bridges TCL’s internal and formal innovation ecosystem with external and informal innovation resources. By leveraging this approach, TCL can effectively navigate formal and informal innovations to enhance its overall innovation capabilities. In essence, the makerspace is repurposed as an intermediary innovation incubator.

The emergence of State-led mass innovation spaces (MIS 众创空间) can be attributed to the trajectory of the MIME policy (Fu, 2021). Notably, several of these types of MISs are financially supported by local governments or state-owned enterprises. The institutionalization and formalization of these spaces embody a nuanced top-down approach that aligns with the country’s economic and soft power policy objectives, while also prioritizing entrepreneurship and potential economic returns. The BeiDou makerspace provides supporting evidence for this hypothesis. Funded by the China National Space Administration, the BeiDou makerspace was established to facilitate the development of relevant products and services based on BeiDou navigation system technologies by external makers and startups. As a component of the national infrastructure, making within this space contributes to the enhancement of the BeiDou application ecosystem, which is critical to national interests. Moreover, new projects and services have the potential to generate profits in the market. Thus, MISs operate with a dual purpose, serving both national interests and market demands.

In the context of China, the makerspace model adopted by enthusiast clubs is relatively infrequent, and a considerable proportion of such models operate “informally”. Scholars have categorized this model as a commons-based peer production community [41], whereby members engage in collaborative efforts to generate novel ideas and innovations by leveraging their shared interests and resources. SZDIY, established in 2009, is one of the pioneering Chinese hackerspaces that emphasizes open source hardware and software development. Members pool their resources to acquire necessary tools, equipment, and space, ensuring shared access to all members. The fundamental principle of Free and Open-source is paramount to the operation of SZDIY, which serves as a local informal hub for open-source technology enthusiasts in Shenzhen. Unlike the previous two models, the enthusiast club is characterized by a more egalitarian structure and informality, with members free to form teams or pursue individual projects. The process of co-creating value and innovation is facilitated by the unimpeded exchange of knowledge and resources. However, this collaborative mechanism is inherently fragile and can only flourish within a relatively egalitarian governance framework. The formalization and institutionalization of commercial or political entities can potentially undermine the efficacy of this model.

Through an examination of the institutionalisation and formalisation process of various types of maker communities in China, a more thorough comprehension of the complex interplay between creative and economic development may be attained. This nuanced understanding can facilitate the identification of crucial factors that contribute to the transformation of maker communities and the broader development of CCIs in China.

### Infrastructuralisation: Making the creative city

This section investigates the impact of state policies on the maker movement in urban cities, with a particular focus on the process of infrastructuralisation of maker activities as part of urban CCIs. The infrastructuralisation process refers to the transformation of informal and loose activities into more structured and formalised ones through the establishment and provision of certain infrastructures, such as technologies, institutions, and policies [42, 43]. The concept of infrastructuralisation highlights the importance of infrastructure development in shaping and changing systems, practices, and power relations. It is a process that can be observed in various social domains, including manufacturing, entertainment, education, and healthcare. For instance, in entertainment, infrastructuralisation can be observed through the development of social media affordances, such as live streaming and virtual meetings. Therefore, the study of infrastructuralisation is crucial in understanding the role of policies and infrastructures in shaping the creative potential of urban CCIs and promoting sustainable urban development.

This study aims to examine the infrastructural effects of MIME policies on the innovative capability of grassroots in addressing urban development challenges, such as economic stagnation, unemployment, and urban transformation. In contrast to Florida’s [44, 45] widely applied concept of creative cities in many Western societies, we aim to provide a more locally rooted understanding of creative cities. Specifically, we examine how MIME policies can contribute to the infrastructuralisation of maker activities and foster the innovative capacity of grassroots in addressing urban challenges.

We chose Shenzhen as the research site to investigate the infrastructuralization process of maker activities in an urban city. We examined the geographic distribution of makerspaces in Shenzhen and observed that these spaces are predominantly situated within the city center. Furthermore, our empirical data demonstrated a noticeable trend of makerspaces co-locating with clusters of CCIs or Science and Technology (S&T) throughout the city. To exemplify this phenomenon, we present the case of Shenzhen Software Park, situated in the Nanshan District’s Hi-tech Zone, a prominent software technology hub in China. During our on-site investigation, we observed the presence of not only the Tencent Creative Space, but also numerous other creative spaces and incubators of varying sizes, including but not limited to BSICIC, TCL, and Shunde, scattered across the park.

Drawing from fieldwork data, we found that this co-location can primarily be attributed to two primary mechanisms: top-down and bottom-up. The top-down mechanism is characterized by the government’s reconstruction of urban spaces and industries through public policies. This is not a novel concept in China, as numerous creative industries parks have emerged nationwide since 2005, following state-led CCIs policies aimed at intentionally clustering CCIs entities into designated locations. This strategy harnesses the creativity/knowledge spillover effects to promote local CCIs development, as noted by Keane [46]. Similarly, the co-location of the maker industry and CCIs in Shenzhen is also driven by public policies, aiming to leverage the creativity/innovation spillover and clustering effects to enhance local innovation capacity and vitality.

On the one hand, it is observed that local CCIs clusters demonstrate proactive efforts to attract makers and makerspaces in response to government initiatives. This assertion is supported by the statement of the manager of the TCL makerspace, as revealed during an interview:

“*We’ve made significant efforts to attract makers and teams as their presence is crucial in our evaluation process by the government. The evaluation decides the level of subsidy and support we receive from the government, which is essential for our growth. Hence, every creative park or cluster is putting in their best efforts to attract makers*.”

*(Interviewee Zhu, 2020)*

On the other hand, it is noteworthy that makers and maker communities are increasingly drawn to establish their presence in these CCIs clusters due to the perceived benefits of lower operating costs, coupled with increased access to a broader range of subsidized innovation infrastructure. This trend is especially pronounced in light of the significant financial challenges that many makers face during the early stages of their innovation journey. By co-locating with other makers in the cluster, these entrepreneurs can share the costs of essential resources, such as equipment and facilities, which would be prohibitively expensive if obtained individually. Moreover, by leveraging the subsidized infrastructure provided by the cluster, makers can gain access to a more extensive range of cutting-edge technology and equipment, which can help to enhance their overall innovation performance. This is corroborated by a growing body of research, which highlights the critical role of CCIs clusters in fostering innovation and promoting regional economic development.

In contrast to the top-down mechanism, the bottom-up mechanism for the formation of maker communities is driven more organically by the makers themselves. Given the need for various innovative infrastructural resources to facilitate their creations, makers tend to gravitate towards locations that offer concentrations of such resources, such as CCIs and Science and Technology (STS) clusters. Our fieldwork has revealed that a significant proportion of makers have backgrounds in STEM fields and work in science and technology industries, leading to early local maker communities predominantly being located near STS clusters.

It is important to note that these CCIs or STS clusters have been the beneficiaries of policies that have promoted their growth, such as rent reductions, reflecting a top-down policy orientation. However, even as later makerspaces located in STS parks have benefitted from these policies, they have also been subject to top-down influences, as previously documented in the literature on the Chinese maker movement [19, 47]. This highlights the significant influence and appropriation of public policies on the Chinese maker movement.

Despite the positive impact of these policies on the co-location effect, the inconsistency and unsustainability of public policies in this regard require further examination. In summary, while top-down policies have played a crucial role in promoting the growth of maker communities, their effectiveness and long-term sustainability remain a subject of debate and inquiry.

## Discussion: Between the center and periphery

In this section, we use the “center and periphery” theory to discuss the three layers analysis of the China’s maker movement. In the initial layers of our investigation, we analysed the strategies utilized by once periphery Chinese makers in navigating the tension between commercialism and criticality to re-locate them to the center of CCIs. Our study highlights the importance of critical introspection and discourse on the role of commodification in shaping the future of maker culture, not just within China, but globally. Moving on to the subsequent layer of our study, we delve into the formalization process of maker communities. By scrutinizing the institutionalization and formalization of diverse types of maker communities in China, we aim to cultivate a more comprehensive understanding of the intricate interplay between creative and economic progress. By applying the center-periphery model to this layer, we can investigate the structural relationship and distribution of power between the advanced center and less-advanced periphery within the formalized maker communities. In the final layer of our study, we examine the impact of governmental policies on the maker movement in urban centers, with a specific emphasis on the infrastructuralization of maker activities as a component of urban CCIs. The center-periphery model enables us to investigate the relationship between the co-location pattern between the makerspaces and local CCIs clusters.

The findings suggest that these transformations have moved the once-peripheral maker culture to the forefront of China’s national CCIs blueprint on the one hand, move China from the global creative peripheral to the center. Similarly, Armstrong et al. [48] have shown how informality plays a significant role in fostering creativity among maker communities in South Africa, while [49] investigation into the coexistence of formal and informal circuits in China’s digital economy provides an insightful analysis of the country’s ongoing digital transformation. These studies, despite their different foci, offer a compelling linkage between the criticality of informality in promoting creativity and the importance of understanding the interplay between formal and informal, center and peripheral in shaping a country’s creative industries development.

## Conclusion

This study explores the emergence and evolution of China’s maker movement and its interplay with the cultural and creative industries (CCIs), utilizing the "center-periphery" theory. By analyzing the maker movement at three levels (individual, organizational, and urban), the study offers a unique perspective on China’s creativity issues. The findings challenge the notion that China is incapable of creativity and innovation due to institutional and ideological influences, demonstrating that an alternative mode of creativity can emerge outside global creative centers.

The study reveals that the Chinese state has effectively developed the maker movement and CCIs through a top-down approach, leading to a transformation characterized by commodification, formalization, and infrastructuralization processes. This approach highlights the attempts to address the unsustainability of CCIs’ innovation and creativity inputs [50], which are crucial to building an "innovative and creative nation." However, policy uncertainty and unsustainability pose challenges to the continued growth of the top-down maker movement. Moreover, the study argues that the maker movement is an effective path for changing the nature of CCIs development models, emphasizing the material aspects of cultural production. However, combining the maker movement with CCIs has also brought some impacts on grassroots innovation culture, raising questions about where to draw the line between intervention and interruption.

This research contributes to the ongoing debate on how China can incorporate the disruptive maker movement into the CCIs development agenda in the Chinese context. The seemingly contradictory combination of critical maker culture and CCIs was appropriated and negotiated by top-down public policies [51]. These policies have supported the development of the maker movement while delineating boundaries and creating collective social, cultural, and techno imaginaries that fundamentally transformed the imported Maker culture into a circumscribed but resilient one.

While the research found that many makers and startups have benefited greatly from such a top-down transformation, policy uncertainty and unsustainability cast a shadow over the continued growth of the top-down maker movement in China. Furthermore, the combination of the innovation and entrepreneurship-oriented maker movement with CCIs has brought many business opportunities but has also had some impacts on grassroots innovation culture. The hard question here is where to draw the line between intervention and interruption. Future research could further explore the downsides and limits of such a top-down approach.

## References

[1] He S. (2019). The creative spatio-temporal fix: Creative and cultural industries development in Shanghai, China. Geoforum, 106, 310–319.

[2] Keane M. (2013). Creative industries in China: Art, design and media. John Wiley & Sons.

[3] Fung A. Y., & Erni J. N. (2013). Cultural clusters and cultural industries in China. Inter-Asia Cultural Studies, 14(4), 644–656.

[4] Shan S. L. (2014). Chinese cultural policy and the cultural industries. City, Culture and Society, 5(3), 115–121.

[5] Hutton W. (2007). The writing on the wall: China and the west in the 21st century. Little, Brown.

[6] O’Connor J, & Gu X. (2014). Creative industry clusters in Shanghai: a success story? International Journal of Cultural Policy: CP, 20(1), 1–20. doi: 10.1080/10286632.2012.740025

[7] Keane M. (2009a). Creative industries in China: four perspectives on social transformation. International journal of cultural policy, 15(4), 431–443.

[8] Zhang X. (2016). The cultural industries in China: A historical overview. In Handbook of cultural and creative industries in China. Edward Elgar Publishing.

[9] Pang L. (2012). Creativity and its discontents: China’s creative industries and intellectual property rights offenses. Duke University Press.

[10] Xinhua News Agency. (2020). China’s cultural digitization and digital culture industrialization presses the "accelerating button". http://www.gov.cn/xinwen/2022-06/18/content_5696617.htm

[11] Flew T., Ren X., & Wang Y. (2019). Creative industries in China: the digital turn. In A research agenda for creative industries. Edward Elgar Publishing.

[12] Keane M. (2016). Handbook of cultural and creative industries in China. Edward Elgar Publishing.

[13] Keane M., & Chen Y. (2019). Entrepreneurial solutionism, characteristic cultural industries and the Chinese dream. International Journal of Cultural Policy, 25(6), 743–755.

[14] National Bureau of Statistics. (2021). Statistical Classification of the Digital Economy and its Core Industries 2021. http://www.stats.gov.cn/tjsj/tjbz/202106/t20210603_1818134.html

[15] Bonet, L., Colbert, F., & Courchesne, A. (2011). From Creative Nations to Creative Cities: An example of center-periphery dynamic in cultural policies.

[16] Hannerz U. (1989). Culture between center and periphery: toward a macroanthropology. Ethnos, 54(3–4), 200–216.

[17] Curtin M., & Sanson K. (2016). Precarious creativity. Global Media, Local Labor, Oakland, CA.

[18] Grodach C., O’Connor J., & Gibson C. (2017). Manufacturing and cultural production: Towards a progressive policy agenda for the cultural economy. City, culture and society, 10, 17–25.

[19] O’Connor J., & Gu X. (2020). Red creative: Culture and modernity in China. Intellect Books.

[20] Millard J., Sorivelle M. N., Deljanin S., Unterfrauner E., & Voigt C. (2018). Is the maker movement contributing to sustainability?. Sustainability, 10(7), 2212.

[21] Mersand S. (2021). The state of makerspace research: A review of the literature. TechTrends, 65(2), 174–186.

[22] Fu P., Sarpong D., & Meissner D. (2021). Recalibrating, reconfiguring, and appropriating innovation: a semantic network analysis of China’s mass innovation and mass entrepreneurship (MIME) initiatives. The Journal of Technology Transfer, 1–18.

[23] Wen W. (2017). Making in China: Is maker culture changing China’s creative landscape?. International Journal of Cultural Studies, 20(4), 343–360.

[24] Bolli M. (2020). Innovators in urban China: Makerspaces and marginality with impact. Urban Planning, 5(ARTICLE), 68–77.

[25] Fu P. (2021). From bottom-up to top-down: governance, institutionalisation, and innovation in Chinese makerspaces. Technology Analysis & Strategic Management, 33(10), 1226–1241.

[26] Dougherty D. (2012). The maker movement. Innovations: Technology, governance, globalization, 7(3), 11–14.

[27] Charmaz K., & Mitchell R. G. (2001). Grounded theory in ethnography. Handbook of ethnography, 160, 174.

[28] Huberman, A. M., & Miles, M. B. (1994). Data management and analysis methods.

[29] LeCompte M. D., & Schensul J. J. (1999). Analyzing & interpreting ethnographic data (Vol. 5). Rowman Altamira.

[30] LeCompte M. D., & Schensul J. J. (2012). Analysis and interpretation of ethnographic data: A mixed methods approach (Vol. 5). Rowman Altamira.

[31] Keane M., & Zhao E. J. (2012). Renegades on the frontier of innovation: The shanzhai grassroots communities of Shenzhen in China’s creative economy. Eurasian Geography and Economics, 53(2), 216–230.

[32] Dong M., & Flowers S. (2016). Exploring innovation in Shanzhai: the case of mobile phones. Asian Journal of Technology Innovation, 24(2), 234–253.

[33] Lindtner S., Greenspan A., & Li D. (2015, August). Designed in Shenzhen: Shanzhai manufacturing and maker entrepreneurs. In Proceedings of the fifth decennial Aarhus conference on critical alternatives (pp. 85–96).

[34] Chubb A. (2015). China’s Shanzhai Culture: ‘Grabism’and the politics of hybridity. Journal of Contemporary China, 24(92), 260–279.

[35] Montgomery L., & Fitzgerald B. (2006). Copyright and the creative industries in China. International Journal of Cultural Studies, 9(3), 407–418.

[36] Deibert R. (2014). DIY citizenship: Critical making and social media. MIT press.

[37] Ratto M. (2011). Critical making: Conceptual and material studies in technology and social life. The information society, 27(4), 252–260.

[38] Barbrook R., & Cameron A. (1996). The californian ideology. Science as culture, 6(1), 44–72.

[39] van Holm E. J. (2017). Makerspaces and local economic development. Economic Development Quarterly, 31(2), 164–173.

[40] Colegrove T. (2013). Editorial board thoughts: libraries as makerspace?. Information Technology and Libraries (Online), 32(1), 2.

[41] Aryan V., Bertling J., & Liedtke C. (2021). Topology, typology, and dynamics of commons-based peer production: On platforms, actors, and innovation in the maker movement. Creativity and innovation management, 30(1), 63–79.

[42] Helmond A., Nieborg D. B., & van der Vlist F. N. (2019). Facebook’s evolution: Development of a platform-as-infrastructure. Internet Histories, 3(2), 123–146.

[43] Kasarda J. D., & Rondinelli D. A. (1998). Innovative infrastructure for agile manufacturers. MIT Sloan Management Review.

[44] Florida R., Mellander C., & Qian H. (2008). Creative China? The University, Human Capital and the Creative Class in Chinese Regional Development.

[45] Florida R. (2005). Cities and the creative class. Routledge.

[46] Keane M. (2009b). Great adaptations: China’s creative clusters and the new social contract. Continuum, 23(2), 221–230.

[47] Fu, P. (2022). The Maker Movement and Creative Industries in China (Doctoral dissertation, Monash University).

[48] Armstrong, C., de Beer, J., Kraemer-Mbula, E., & Ellis, M. (2018). Institutionalisation and informal innovation in South African maker communities.

[49] Zhao E. J. (2019). Digital China’s informal circuits: Platforms, labour and governance. Routledge.

[50] Saunders T., & Kingsley J. (2016). Made in China: Makerspaces and the search for mass innovation. London: Nesta.

[51] Liao C., & Fu P. (2022). Love your idol in a ‘cleaned’way: Fans, fundraising platform, and fandom governance in China. Media International Australia, 1329878X221095580.

